# Is Childhood Disadvantage Temporary or Permanent? Evidence From the Study on Global Ageing and Adult Health in Ghana

**DOI:** 10.1093/geroni/igaa057.1078

**Published:** 2020-12-16

**Authors:** Paul Ayernor

**Affiliations:** City University of New York-CUNY, Staten Island, New York, United States

## Abstract

The paper assesses whether childhood socioeconomic status have a temporary or permanent effect on adult health status and well-being. The study uses cumulative inequality theory to explain disparity in health status and well-being at older ages in Ghana. Data comes from the 2007-2008 World Health Organization global study of ageing in Ghana (SAGE). The study utilizes wave 1 of the data, with retrospective questions about early childhood socioeconomic status. The study uses ordinal logistic regression models to assess the relationship between childhood socioeconomic status and self-report health on one hand and wellbeing on the another. The results show that father’s education is a significant predictor of health status and wellbeing at older ages. Specifically, the odds of reporting good and moderate health status and wellbeing are 1.29 and 2.22 times higher among older adults whose fathers have primary education or higher. As expected, the odds of reporting moderate or good health status and wellbeing decrease with increasing age and also for women. In terms of interaction effects, those aged 60-69 years whose fathers have primary education are less likely to report good and moderate health. In contrast, those who are aged 70-79 years old and have fathers with secondary or higher education are 2.51 times more likely to report good and moderate wellbeing. There is strong evidence of compensation among those who keep once or twice contact with social ties.

